# Applications and Mechanisms of Ionic Liquids in Whole-Cell Biotransformation

**DOI:** 10.3390/ijms150712196

**Published:** 2014-07-09

**Authors:** Lin-Lin Fan, Hong-Ji Li, Qi-He Chen

**Affiliations:** 1 Department of Food Science and Nutrition, Zhejiang University, Hangzhou 310058, China; E-Mails: 11213025@zju.edu.cn (L.-L.F.); 21313060@zju.edu.cn (H.-J.L.); 2 Fuli Institute of Food Science, Zhejiang University, Hangzhou 310058, China

**Keywords:** ionic liquids (ILs), whole-cell catalysis, mechanism, biotransformation

## Abstract

Ionic liquids (ILs), entirely composed of cations and anions, are liquid solvents at room temperature. They are interesting due to their low vapor pressure, high polarity and thermostability, and also for the possibility to fine-tune their physicochemical properties through modification of the chemical structures of their cations or anions. In recent years, ILs have been widely used in biotechnological fields involving whole-cell biotransformations of biodiesel or biomass, and organic compound synthesis with cells. Research studies in these fields have increased from the past decades and compared to the typical solvents, ILs are the most promising alternative solvents for cell biotransformations. However, there are increasing limitations and new challenges in whole-cell biotransformations with ILs. There is little understanding of the mechanisms of ILs’ interactions with cells, and much remains to be clarified. Further investigations are required to overcome the drawbacks of their applications and to broaden their application spectrum. This work mainly reviews the applications of ILs in whole-cell biotransformations, and the possible mechanisms of ILs in microbial cell biotransformation are proposed and discussed.

## 1. Introduction

Ionic liquids (ILs) are collectively known as organic salts, which consist of ions and are liquids at room temperature [1]. They have attracted great interest as the high-tech reaction media of the future with distinct properties, such as negligible vapor pressure, low melting point, varying viscosities, larger electrochemical window and thermal stability [2,3,4,5]. These physical properties mostly depend on both the type of cation/anion and the alkyl chains on the anions [6,7]. Additionally, the polarity, hydrophobicity and solvent miscibility behaviour of ILs can be finely tuned through appropriate modification of the cation and anion [8,9] so as to meet the requirements of applications. In recent years, ILs have been widely used in analytical chemistry [10], biodiesel and biomass [11,12,13,14], and biotechnology, particularly in biocatalytic reactions which can be performed by using isolated enzymes or whole cells [15,16]. According to studies, there are several role models in biocatalysis process with ILs. So far, ILs have been used as pure solvents, as co-solvents in the aqueous phase, or as biphasic systems together with other solvents and even with combination of polymer for the enhancement of both chemical and enzymatic reactions [17,18,19,20,21]. For example, (BMIM)(PF_6_) and (BMIM)((CF_3_SO_2_)_2_N) were usually used as pure solvents, and also as co-solvents in biphasic systems, while (BMIM)(BF_4_) or (MMIM)(MeSO_4_) was a co-solvent in the aqueous phase [22]. Many enzymatic reactions, such as lipase catalyzed reaction, transesterification, hydrolysis and condensation, and ammoniolysis reactions have successfully proceeded [23,24,25,26]. In these processes, the activity and stability of several enzymes have been reported to be increased or modulated in the presence of ILs [23,27,28,29] Meanwhile, an increasing number of investigations have concerned applications of ILs in analytical and biological sciences [10,30,31]. Extraction, separation, and purification of organic compounds [32], amino acids [33], as well as DNAs [34] from complex matrixes are also performed by employing ILs, providing potentials for expanding applications in life science.

However, reports on whole-cell biocatalysts with ILs are relatively few. Generally, in the whole-cell reactions with an aqueous medium, hydrophobic ILs are used as a second liquid phase. The microbial cells are usually dispersed in the water phase, and the substrate and products are mostly kept in the ILs phase. Thus, ILs can act as a hydrophobic product reservoir to deliver the substrate into the aqueous phase [35], which can make reactions more efficient than those systems without ILs. Meanwhile, ILs have an effect on the cells as a part of fermentation media. Whole-cell biocatalysts exhibit an increased stability and additional benefit in comparison to the isolated enzymes [15]. Unfortunately, ILs are reported to have toxic effects towards bacteria, yeasts and fungi [36,37,38,39], which are limitations in the biotransformation applications. Therefore, an understanding of the mechanisms of the effects of ILs on microorganism cells are necessary to reveal possibilities for enhancing the biotransformation yield, and this research area has thus attracted much attention and interest in past years. Herein, the applications of ILs in whole-cell biotransformations are summarized in this review. The issues that surround this field, including the characteristics involved in whole-cell biotransformatins, the activity and stability changes of enzymes in the presence of ILs and the interactions of ILs towards cells, are discussed. Furthermore, possible mechanisms of ILs in microorganism cells are proposed.

## 2. Characteristics of Ionic Liquids for Whole-Cell Catalysis

**Figure 1:**
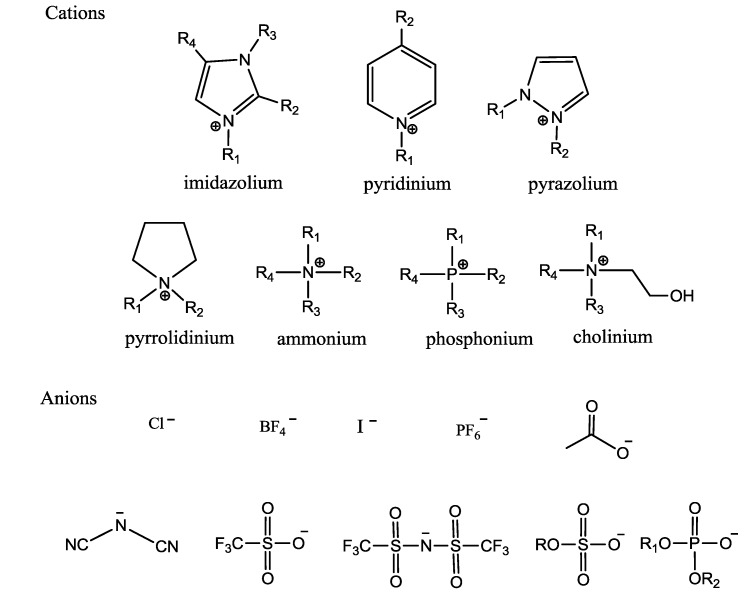
Main cations and anions described in studies.

Various kinds of anions and cations available to be ILs have been studied so far. Newly designed ILs are developing into green solvents with the range of their applications expanding enormously. In terms of prediction [40], there are approximately one trillion (10^18^) accessible ILs. However, the ILs that can be used for biotransformation are few. Some structures of cations and anions commonly used in whole-cell biocatalysis are summarized in Figure 1.

It is clear that the cations are mainly asymmetric substances with characteristics of large volume, weak intermolecular interaction and lower charge density, such as 1-butyl-3-methylimidazolium (BMIM^−^), 1-octyl-3-methylimidazolium (OMIM^−^), 1-butylimidazolium (HMIM^−^), 1-ethyl-3-methylimidazolium (EMIM^−^), 1-hexyl-1-methylpyrrolidinium (HMPL^−^), *etc.* Imidazole-, pyridine-, pyrrole-, amino-, matte-, phosphine- and their derivatives, especially the imidazolium-based salts are mostly investigated. The anions are classes of multi-core and single-core anion, and the latter is applied widely. The common anions of ILs are BF_4_^−^, PF_6_^−^, Tf_2_N^−^, ZnCl_3_^−^, CuCl_2_^−^, N(CF_3_SO_2_)_2_^−^, N(FSO_2_)_2_^−^, CF_3_CO_2_^−^, CF_3_SO_3_^−^, MeSO_3_^−^, Al_2_Cl_7_^−^, Au_2_Cl_7_^−^, Fe_2_Cl_7_^−^, and Sb_2_F_11_^−^, *etc.* [41].

The composition and structure type are the key factors to physicochemical properties of ILs, togther with biological performances. Moreover, their tunable properties vary with modified structure [42,43]. Compared with the conventional organic solvents, ILs are beneficial for applications and they are indispensable alternatives to organic solvents. Although the properties of ILs have been described within a number of publications [37,43,44], the obvious properties which are important for whole-cell biotransformation are briefly discussed in this section to expound the structural relationship of ILs to their properties.

### 2.1. The Melting Point of ILs

ILs possess a low melting point which is determined by the types of cation and anion, ranging from 0 to 100 °C. It is much lower in the case of the weaker molecular interaction, lower structural symmetry and uniformly distributed cation/anion charges. Additionally, ILs have a broad temperature window at or below 300 °C, in which condition they remain as liquids. This is superior to classical solvents. However, recent studies pointed that the melting point of ILs was uncertain due to the potential presence of impurities [45]. In light of the importance of temperature to microorganism growth conditions, ILs with an equal melting point are essential to be developed and employed.

### 2.2. The Viscosity of ILs

The viscosity is a remarkable property for IL use. Most ILs are much more viscous than organic solvents. For example, (BMIM)(BF_4_) has a viscosity similar to ethylene glycol [19.6 Centi Poise (cP) at 25 °C, and much higher than that of water (0.9 cP)], methanol (0.5 cP) or toluene (0.6 cP); (BMIM)(PF_6_) is 20 times more viscous than *n*-hexadecane [6,46]. The viscosity of ILs is usually affected by strong intermolecular forces between solvent molecules, the alkyl chain length, organic cosolvents and water. For ILs, the strong forces are their inherent charge-charge interactions, and van der Waals forces also exist between molecules. The reducing van der Waals interactions can cause slightly lower viscosity by reducing the surface area of the molecules [46]. Similarly, ILs are less viscous in the presence of water or organic cosolvents, because the hydrogen bonding and the strength of its van der Waals interactions are lower. On the other hand, ILs with longer alkyl chains exhibit much higher viscosity than those with shorter chains on the cations [47]. Since viscous IL-containing medium makes some operations including mixing, filtrating, and other mass transfer more difficult, and the metabolism of cells would be inhibited, less viscous ILs need to be designed for wide applications.

### 2.3. The Density of ILs

The density of ILs (ranging from 1.1 to 1.6 g·cm^−3^) is generally larger than typical solvents or water. It is noted that the higher density is conductive for ILs to be separated from aqueous cell-containing phases [6]. Very slight differences of structure can result in changes of density. In many cases, the density decreases by the reduced volume of the anion or the increasing alkyl chain length of the cation. It is related to mass transfer in reaction systems, and ILs with designed structure and density are beneficial for cell biotransformation.

### 2.4. The Polarity of ILs

Polarity is one of the most important properties of ILs. In general, solvents with high polarity can affect the enzymes and microbial cells, resulting in enzyme activity reduction or deactivation. On the contrary, ILs with high polarity can make enzymes stable and selective [22,48], leading to an increased reaction rate. Since ILs have the ability to dissolve many polar or nonpolar substances, they can be used in both hydrophilic substrate reactions and hydrophobic substrate reactions. However, it is difficult to define the polarity of one kind of ILs, because different results can be observed when determined based on the shift of the charge-transfer absorption band of a solvatochromic probe [49,50,51], such as Reichardt’s dye and Nile red dye. Methods of partition and fluorescence probe have also been used for polarity determination [52]. All the results revealed that ILs are polar with a polarity between water and some alcohols. However, it varies with the structure changes of cation/anion, and decreases with an increase of the alkyl chain length [53]. In cell biotransformation, the polarity is essential to mass transfer. An IL with shorter alkyl chains on the cation has higher polarity and a lower viscosity at the same time. This IL is helpful to accelerate the catalysis reactions. But it is uncertain whether the viscosity and the polarity changes account for the main reason of the changing reaction rate together or respectively. The viscosity change may be the key factor, because the viscosity is affected by the alkyl chain length more. Additionally, the polarity of ILs is sometimes susceptible to water and temperature [54]. And the presence of water can affect the properties of ILs. However, the solubility of water in ILs is various depending on the types of anions. This affect should be taken into consideration when ILs are applied to biotransformation in culture broth with cells.

## 3. Whole-Cell Biocatalytic Transformations in Ionic Liquids

Biotransformation provides a green method which uses natural or engineered metabolic pathways for production of high value-added chemicals from renewable feedstocks [55,56,57]. Unfortunately, the organic solvents used in the process may be toxic to cells and restrict the enzyme activity or selectivity, leading to decreased productivity and uneconomic processing. For this reason, alternative ILs for reaction media, and for a reservior in whole cell biotransformations is indispensible and there is an increasing considerable interest in using ILs as the reaction medium for biotransformation [58].

Reports of ILs application in whole-cell biotransformation including application for biodiesel synthesis with cells [59,60], enzyme production [61] or substrate and synthesis of organic compounds in microbial cells [62,63,64,65], have increased from the past decade; various kinds of yeasts, bacteria and fungi have been researched and developed to whole-cell biosynthesis. Some examples were summarized in Table 1.

**Table 1:** Examples of applications of ILs in whole-cell biotransformations.

Microorganism	ILs			Biocatalysis Reaction		Improvement		Reference	
*Penicillium purpurogenum* Li-3	(BMIM)(BF_4_)			Biosynthesis of glycyrrhetic acid 3-*O*-mono-β-d-glucuronide (GAMG)		A yield of 2.62 g·L^−1^ after 62 h in IL co-solvent medium compared to 2.34 g·L^−1^ after 72 h in buffer medium.		[17]	
*Rhodotorula glutinis* ATCC201718	(DEME)(Tf_2_N) (BMIM)(PF_6_) (HMIM)(PF_6_) (OMIN)(PF_6_) *etc.*			Hydrolysis reaction of racemic 1,2-epoxyhexane		The high enantiomeric ratio of (*R*)-diol (E > 100) without significant decrease in the reactivity was accomplished by adding 1-heptanol in minute amounts to dodecane.		[66]	
*Saccharomyces cerevisiae* *Candida albicans* *Rhodotorula glutinis* *Geotrichum candidum* *Micrococcus luteus*	(BMIM)(BF_6_)			Reductions of (Z)-C_6_H_5_CH=CXC (=O)CH_3_ (X = Cl, Br)		Better diastereoselectivity and enantiose-lectivity than in pure water.		[35]	
*Penicillium purpurogenum* Li-3 (w-PGUS) *Escherichia coli* BL21 *Pichia pastoris* GS115	(BMIM)(BF_6_)			Hydrolysis of glycyrrhizin (GL) to glycyrrhetic acid 3-*O*-mono-β-d-glucuronide (GAMG)		The 60 g·L^−1^ (1.23 U/g) cell concentration, a GAMG yield of 87.63% was achieved after 60 h.		[67]	
*Acetobacter* sp. CCTCC M209061	(C_2_OHMIM)(NO_3_)			Reduction of 4-(trimethylsilyl)-3-butyn-2-one reduction to (*R*)-4-(trimethylsilyl)-3-butyn-2-ol		The initial reaction rate, the maximum yield and the product *e.e.* were 14.0 mmol·min^−1^·(g·cell)^−1^, 91%, and 499%, respectively.		[68]	
*Aspergillus ochraceus*	(C_3_MIM)(PF_6_)			Reduction of 11α hydroxylation of 16α,17-epoxyprogesterone (HEP)		The substrate conversion reached 90% with a substrate concentration of 20 g·L^−1^ under the selected conditions.		[69]	
*Escherichia coli*	((EO_2_E)MPL)(NTF) (HMPL)(NTF) (HPYR)(NTF) ((NEMM)EO_2_E)(NTF)			Reduction of 2-octanone to (*R*)-2-octanol		Various ionic liquids can be used for this reaction and the ionic liquid volume fractions are up to 40%.		[70]	
*Escherichia coli*	(HMPL)(NTF)			The asymmetric reduction of 2-octanone to (*R*)-2-octanol		The average conversion was 98.5 (±0.7)%, and enantiomeric excesses were constant at values ≥99.5% (*R*). A total of 999 (±6) g _(*R*)-2-octanol_·L^−1^_IL_ was produced.		[42]	
*Rhizopus nigricans*	(BMIM)(PF_6_) (BMIM)(NTf_2_)			11α-Hydroxylation of 16α,17-epoxyprogesterone		The conversion was greatly increased to above 90% at 18 g·L^−1^ feeding concentration.		[71]	

### 3.1. Whole-Cell Biotranformation in Ionic Liquids by Bacterium

**Figure 2:**
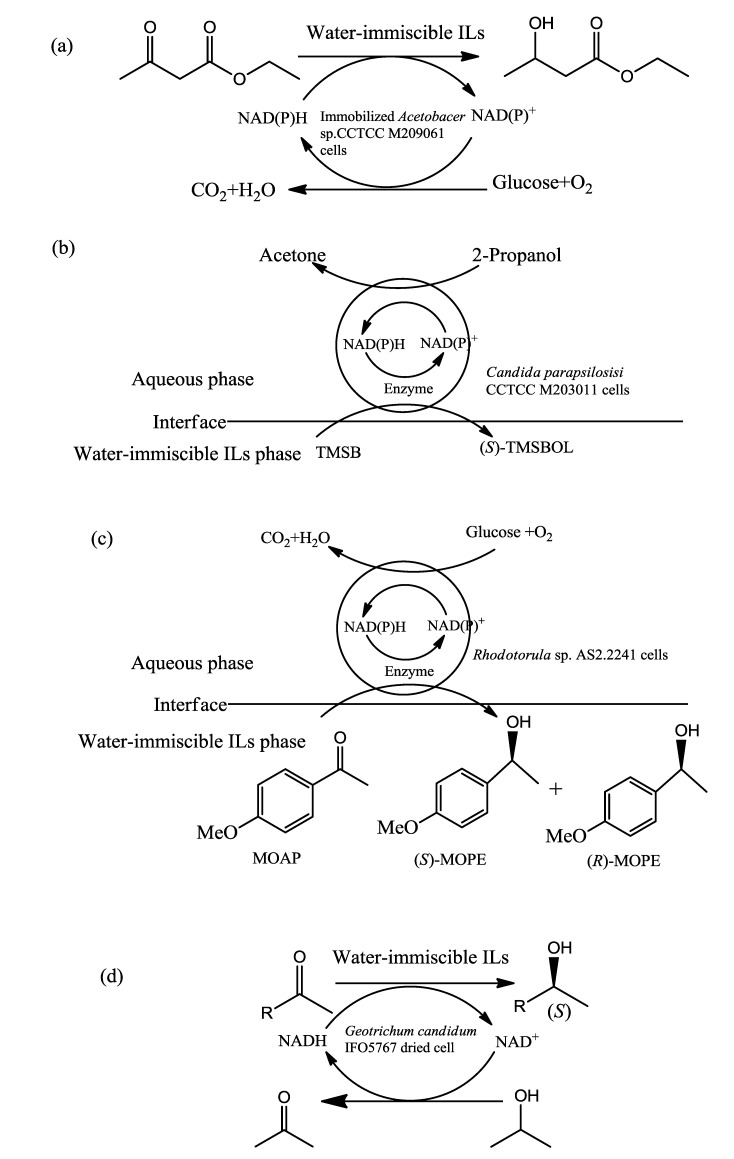
Examples of biotransformation from microorganism cells with ILs. (**a**) The asymmetric reduction of ethyl acetoacetate (EAA) catalyzed by immobilized *Acetobacter* sp. CCTCC M209061 cells; (**b**) The bioreduction of 4-(trimethylsilyl)-3-butyn-2-one (TMSB) to (*S*)-4-(trimethylsilyl)-3-butyn-2-ol (*S*)-TMSBOL with immobilized *Candida* *parapsilosis* CCTCC M203011 cells in water-immiscible IL/buffer biphasic systems; (**c**) The biocatalytic asymmetric reduction of 4'-methoxyacetophenone (MOAP) catalyzed by *Rhodotorula* sp. AS2.2241 cells; (**d**) Asymmetric reduction of ketone and recycling of coenzyme catalyzed by *Geotrichum candidum* dried cell containing alcohol dehydrogenases.

There have been many successful examples of using ILs for whole-cell biotransformations from bacteria so far. Cull *et al.* [72] have first reported the results on the use of an IL as a reaction phase for product biotransformations. The (BMIM)(PF_6_) was used in a two-phase system for the hydration of 1, 3-cyanobenzene catalysed by nitrile hydratase from *Rhodococcus* R312 to produce 3-cyanobenzamide and 3-cyanobenzoic acid. The substrate had better solubility in IL, which acted as a reservoir, than in water. The addition of (BMIM)(PF_6_) increased the specific activity of *Rhodococcus* R312 for the product synthesis, and a slightly higher yield was finally reached. Fewer cells aggregated due to the presence of IL, which was found to be advantageous for the separation of the two phases on completion of the reaction. The *Rhodococcus ruber* was also used successfully for the biocatalytic reduction of ketones in bi- and monophasic IL/buffer systems. The reaction was catalysed by the alcohol dehydrogenase ADH-‘A’ from the cells via hydrogen transfer. In the reaction, density of the (BMIM)(PF_6_) is above 1.2 kg·L^−1^ and viscosity is below 400 mm^2^·s^−1^, which can secure effective phase separation and avoid mass transfer limitations. Thus we can see that the density and viscosity of ILs play key roles in whole-cell biotransformations. Besides, the (BMIM)(Tf_2_N) exhibits very good solvent properties for 4-chloroacetophenone and its benzyl alcohol reduction product from *Lactobacillus kefir* cells, and there were no destructive effects observed on the cell membranes of *L. kefir* [73]. The recombined or immobilized bacterial cells have also been observed to be conductive to the catalysis process. The immobilized *Acetobacter* sp. CCTCC M209061 cells were capable of catalyzing the asymmetric reduction of ethyl acetoacetate (EAA) in the various IL-based biphasic systems with a high product enantiomeric excess (*e.e.*) of above 99% [74] (Figure 2a). The recombinant *Escherichia coli* strain was active in the presence of (BMPL)(NTf_2_) or (BMIM)(PF_6_) phases and could convert benzaldehyde and cyanide into mandelic acid and mandeloamide. The cells were slightly more effective in the presence of (BMPL)(NTf_2_) than in the presence of (BMIM)(PF_6_) [62].

Water-immiscible imidazolium-based salts are the most investigated, and are found to be suitable solvents for bacterium to carry out biotransformation involving non-polar toxic substrates. However, the potential toxicity of ILs towards microorganisms is particularly taken into consideration in the case of biotechnological applications [6]. Various microorganisms used for biosynthesis with ILs were investigated. For example, water immiscible ILs (BMIM)(PF_6_), (BMIM)(Tf_2_N) and (OMA)(Tf_2_N) are shown to have no damaging effects on the cell membranes of *Escherichia coli* and *Saccharomyces cerevisiae* [75]. (BMIM)(BF_4_) and (EMIM)(EtSO_4_) inhibited the growth of *Clostridium sporogenes* which is an extremely versatile biocatalyst for the transformation of nitrogen-containing precursors to chiral and achiral amines. By contrast, water-miscible IL (EtOHNMe_3_)(Me_2_PO_4_) increased the growth rate of *C. sporogenes* by as much as 28%, suggesting that the IL has either been metabolized or increased the availability of nutrients [76]. Thus, water-miscible ILs may deliver water insoluble substrates into cells for biotransformation. However, they are relatively less used in whole-cell biotransformations, possibly due to their toxicity to most cells [77,78]. Interestingly, ILs with moderate toxicity are found to be suitable for biotransformations [76,79], but the mechanism of these types of ILs with bacteria is not clear.

### 3.2. Whole-Cell Biotranformation in Ionic Liquids by Yeasts

Most yeast are able to convert substrates into valuable compounds with high efficiency. In the past decades, different yeasts or recombined yeasts have been used for the asymmetric reduction of ketones or biomass with ILs.

The strain *Saccharomyces cerevisiae* is the most studied microorganism which has a good biocompatibility with an aqueous two phase system. This characteristic is specially employed in extract fermentation. Furthermore, this yeast is able to synthesize alcohols and ketones of industrial interest in the presence of ILs [64,65,75,80]. For instance, Howarth *et al.* compared yeast-mediated ketone production in (BMIM)(PF_6_) with that in an organic solvent system and observed that the reactivity in IL varied considerably with the substrate structure. The cells in IL biphasic system exhibited less aggregation than in a water-toluene system [81]. Likewise, it is advantageous for the separation of the product and efficient conversion.

Thus yeasts always carried out a good performance of the bioconversion with IL, which may be due to the excellent solvent properties of the IL for substrate and product, and its good biocompatibility with the cells. An example is that the synthesis of (*S*)-TMSBOL is successfully conducted with high yield and excellent product *e.e.* by means of the biocatalytic asymmetric reduction of TMSB using immobilized *Candida parapsilosis* CCTCC M203011 cells in water-immiscible IL-based biphasic systems (Figure 2b). Of all the various tested ILs, the best results were obsevered in the presence of (C_4_MIM)(PF_6_) which exhibited significant effects on the bioconversion [82]. Moreover, the immobilized *Rhodotorula* sp. AS2.2241 was used as a biocatalyst for asymmetric reduction of 4'-methoxyacetophenone (MOAP) to enantiopure (*S*)-1-(4-methoxyphenyl) ethanol (*S*)-MOPE (Figure 2c). The water-immiscible IL (C_4_MIM)(PF_6_) can markedly enhance the efficiency of MOAP reduction to enantiopure (*S*)-MOPE mediated by *Rhodotorula* sp. AS2.2241 cells [83]. Another promising application of using yeasts cell for biotransformation in IL-based system is for enantioselective hydrolysis reaction of racepoxide in order to obtain enantiopure compounds. Matsumoto *et al.* [66] carried out the kinetic resolution of racemic 1,2-epoxyhexane under a hydrophobic solvent/buffer two-liquid phase system using the yeast *Rhodotorula glutinis* ATCC 201718 biocatalyst, and various organic solvents and ILs were used as solvents in this system. [BF_4_]-anion and [CF_3_SO_3_]-anion deactivated the enzyme in the cell membrane which may be affected by the raising of hydrophobicity with the increase in alkyl chain length of ILs; the reactivity of enzyme followed the distribution of ILs from buffer to cell membrane. The electrostatic interaction occurred between the charged molecule consisting of cell membrane and the IL molecule. Therefore, the structures of ILs play an important role in the enzyme activity of cell membranes. Thus, to design and choose suitable ILs is important for the whole-cell bioconversion application.

### 3.3. Whole-Cell Biotranformation in Ionic Liquids by Fungi

Few fungi are employed for conversion with ILs. This may be attributed to the mycelium formation during fermentation. However, the fungi have potential for biodiesel, ketones and chiral alcohol productions and some examples are presented below.

Various ILs were used to enhance the activity of *Penicillium purpurogenum* Li-3 (w-PGUS) cells and alginate gel biocatalyst by Kaleem *et al*. [17]. They were found the best polymer for the biotransformation of glycyrrhizin (GL) into glycyrrhetic acid 3-*O*-mono-β-d-glucuronide (GAMG). The (BMIM)(BF_4_) had an ascending effect on the GL catalysis at the low volumetric ratio and exhibited the positive impact of IL on the biotransformation rate. But while above 30% IL/buffer ratio, the reaction rate decreased dramatically.This is ascribed to the damaging effects of excess ILs on cells. For enzymatic biodiesel production from plant oil hydrolysates, an *Aspergillus oryzae* whole-cell biocatalyst that expresses *Candida antarctica* lipase B (r-CALB) with high esterification activity was developed [84]. Arai *et al.* [85] have investigated biodiesel production in ILs by lipase-producing *Rhizopus oryzae* and *Aspergillus oryzae* as whole-cell biocatalysts. The cross-linking of whole-cell biocatalysts with glutaraldehyde proved to be effective for the stabilization of biocatalysts in biodiesel fuel production in ILs.

Tomoko *et al.* [86] studied the asymmetric reduction of ketones by *Geotrichum candidum* in ILs (Figure 2d). For the case of (BMIM)(PF_6_), the yield was improved because the surface area between the IL and water layers was increased. The cells enzyme was observed to retain a stabilizing effect by keeping water around it as (EMIM)(BF_4_) existed, on which condition the reaction yield was improved. Additionally, the immobilized *Geotrichum candidum* was reported to be applied into the enantioselective oxidation of alcohols to produce chiral alcohols in the ILs [87]; it was shown that the reactions were carried out smoothly with excellent enantioselectivity when the cells were immobilized on water-absorbing polymer containing water. So the water layer around the cell is necessary for the reaction to proceed. Our research team has previously applied ILs for betulinic acid production from betulin biotransformation by *Armillaria luteo-virens* *Sacc* ZJUQH100-6 cells. It was found that the addition of (EMIM)(BF_4_) in hexane-containing reaction medium gave rise to better betulinic acid formation with less reaction time compared with other ILs used [88]. Moreover, the effects of ILs on cells or enzymes have been discussed.

## 4. Interaction Mechanisms of Ionic Liquids with Cells in Whole-Cell Biotransformation

**Figure 3:**
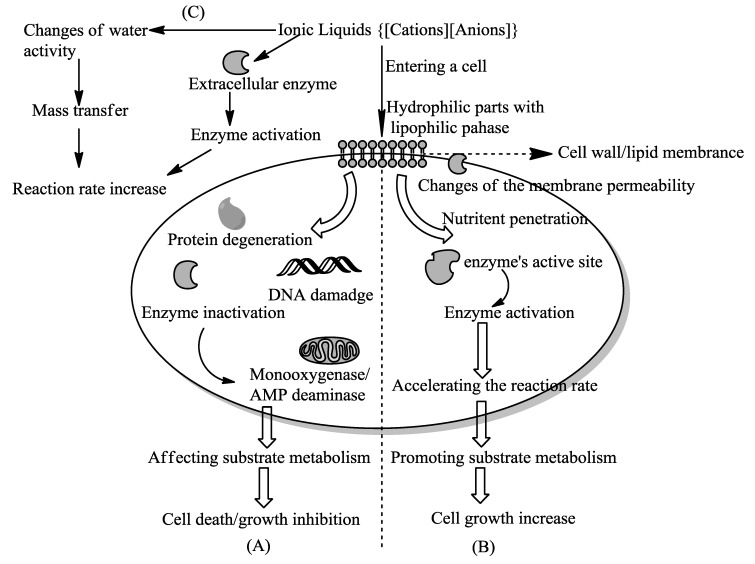
Possible interaction mechanisms of ionic liquids with cells in whole-cell biotransformation. (**A**) Some ILs may affect the cell microstructure and lead to cell growth inhibition or cell death; (**B**) The pathway describes an increase of cell growth in the presence of ILs; (**C**) Some ILs may accelerate the mass transfer and reaction rate.

In the whole-cell biotransformation, ILs used in the medium have inevitable effects on the microorganism cells. Some effects are positive to reactions, and some not. The mechanisms are discussed in this section, and a possible pathway is summarized in Figure 3.

### 4.1. To Regulate The Cell Growth

In terms of the influences of ILs on cell growth, there are potentials for an inhibition to cell growth, an increase of cell growth or an inhibition of the metabolism. The inhibitions of cells are most attributed to IL toxicity, which is investigated as a key drawback for cell biotransformations in abundant studies [36,45,65,89,90,91]. Generally, the cells of microorganisms basically consists of a cell wall and one or two lipid membranes. It is supposed that the hydrophilic parts of the ILs are dissolved in the aqueous phase, with some hydrophobic parts fusing with the lipophilic phase. When entering a cell, they may affect intracellular metabolism via the cell wall and/or the outer membrane. Moreover, the inhibition increases with the increasing length of the alkyl chains [92]. Some ILs increased the growth rate of cells [76], suggesting that they either increased the availability of nutrients or were metabolized as nutrients. Furthermore, a few of the toxic ILs have been reported to be suitable for the whole-cell biotransformations and for production improvement [61]. In the process, both side reactions and unproductive substrates may be suppressed with the inhibited cell growth in ILs.

### 4.2. To Enhance the Reaction Rate

In some cases, the reaction rate is highly enhanced with water miscible ILs as a solvent system. It could be attributed to the hydrophilic nature of ILs, which might interact with the charged groups on substrate and interact with hydrogen or covalent bonding, and transfer electric charge of the cell membrane effectively. This leads to changes in the ionic state of cell membrane and affects membrane permeability [88], allowing the substrate to permeate through the cell membrane more easily. As reported earlier, the polarity of ILs is a key property for whole-cell catalysis. In the case of polar and hydrophobic ILs, such as (BMIM)(PF_6_), the reaction rate increases. It was reported that the increasing hydrophobicity of ILs can lead to an improvement in water activity around the protein, promoting the enzyme action by enhancement of free water molecules [88]. The inclusion of the enzyme in the IL matrix may drive the protein toward a more active conformation for expressing its catalytic activity. Thus, water in an IL-based system plays an important role in enzyme activity improvement. It is noted that the high concentration of the ions in the reaction system could retard the enzymatic activity by an inhibition on the enzyme's active site. Thus, adding water can improve the enzyme activity and accelerate the mass transfer of substrate because of the decreasing viscosity of the reaction medium, but meanwhile the dissolved enzymes show low catalytic activity due to their conformational changes [93]. In short, the enhancement of biotransformation rate in IL-based medium could be ascribed to the increase of the mass transfer and activation of the enzymatic activity [25]. It is also suggested that the toxic ILs used for biotransformation can enhance the product yields and reaction rate. These ILs are inhibitors of cellular respiration processes and thus favor the shunting of NADH and O_2_ towards the overexpressed biocatalytic process [61]. This involves the structures of cations. Thus, it will be possible to design or identify completely biocompatible ILs through optimization of the combinations of anion and cation. That the structure of ILs is stable in water or fermentation medium is necessary in most whole-cell biotransformations.

## 5. Future Perspectives and Conclusions

ILs have been widely used in biocatalysis due to their unique properties. Whole-cells are employed with ILs in the fields of fine chemical, medicine and chemical reagent synthesis, which has development potential. However, the range of ILs suitable for biocatalytic whole-cell applications is still limited. Some problems remain unresolved and need to be investigated further.

### 5.1. Microstructure Changes of Cells in ILs

The mechanism of ILs acting on cells in biotransformation is increasingly studied, but is uncertain due to various effects of IL-based systems on different cells, and thus questions remain open. ILs are usually referred to as substrates which can maintain the integrity of the cell membrane to avoid an outflow of the intracellular cofactors and accelerate the mass transfer. However, as synthesized chemicals, ILs can display many molecular actions modes through interaction with biomacromolecules of cells, such as phospholipids, proteins and DNA [94]. Mostly, the actions can potentially cause disorder of particular metabolic pathways in microbial cells, including changing the membrane permeability, affecting transport proteins, enzyme activity inhibition, and even DNA damage. Studies have focused on these molecular mechanisms with the cytochrome P450 assay [95,96] and the adenosine monophosphate (AMP) deaminase assay [97]. The views discussed above are debatable; the influence of different anion and cation groups on cell membrane potential, intracellular enzymes and even the substrate metabolism have not been investigated thoroughly up to now. For instance, it is known that the chain length of the cation strongly affects the enzyme activity, probably because of the binding to the lipophilic active site, but the effect of the anion is still unclear. We assumed that the optimized ILs and ratio in a reaction system can improve the biotransformation due to the positive interactions with the cell membrance and cell enzymes as mentioned above. To elucidate the mechanism of ILs in cells, strategies and biological techniques, such as transcriptome sequencing analysis and proteomic analysis joined with metabonomics analysis, should be employed. Moreover, the quantitative structure–activity relationships (QSAR) model has been used to predict the functions of ILs [98,99]. Thus, potential functional models of ILs with cells, based on structures, can be effectively estimated. Multidimensional detection means will be applied extensively.

### 5.2. Safety Evaluations of the ILs in Whole-Cell Biotransformations

ILs are generally referred to be “green” solvents. But there is an argument that ILs are inadequate to be sustainable chemical products because of their uncertain toxicity to cells, biodegradability and bioaccumulation [100]. Safety evaluations of ILs have been made in enormous studies [38,39,101,102,103,104], but there are still uncertainties. The problem is that the evaluation of cations and anions are separate. Thus, it is difficult to assess the safety of ILs [100]. However, the hydrophobic ILs with biodegradability are needed primarily, so it is necessary for ILs to be designed to be more biodegradable and with more hydrophobic anions.

Further, it is necessary to design ILs with better performance functions, such as thermostability, operability and recycling to expand their scope of applications. But there are a few problems needing to be addressed before the newly designed ILs are put into use. The new types need to be identified in view of the safety to cells. In the absence of clear structure-activity relationships and a sufficient body of data that QSAR needs for predicting the biocompatibility and hydrophobicity of ILs, the effects on cells or the behavior of substances in the biphasic reaction are difficult. To choose ILs suitable for a given process or to screen microorganisms for the known synthesis where a higher-yield or green process is required remains challenging. Fortunately, to recombine cells with IL resistance established by an inner membrane transporter and regulated by an IL-inducible repressor has become possible [105], and it is important and urgent to identify an IL-resistance mechanism from IL-tolerant microbes. All in all, in whole-cell biotransformation, ILs may become key environmental systems for intracellular enzyme reactions. Given the cost and benefit, less expensive ILs are expected to be applied to industry, and those ILs with no hazards to the environment and a safe quality will be chosen first for future applications. IL-action mechanisms in whole-cell biotransformation need to be clarified to accelerate progress towards effective bioconversion of renewable value-added chemicals.
